# The Incidence of Coronary Heart Disease and the Population Attributable Fraction of Its Risk Factors in Tehran: A 10-Year Population-Based Cohort Study

**DOI:** 10.1371/journal.pone.0105804

**Published:** 2014-08-27

**Authors:** Davood Khalili, Farhad Haj Sheikholeslami, Mahmood Bakhtiyari, Fereidoun Azizi, Amir Abbas Momenan, Farzad Hadaegh

**Affiliations:** 1 Prevention of Metabolic Disorders Research Center, Research Institute for Endocrine Sciences, Shahid Beheshti University of Medical Sciences, Tehran, Iran; 2 Department of Epidemiology, School of Public Health, Shahid Beheshti University of Medical Sciences, Tehran, Iran; 3 Department of Epidemiology and Biostatistics, School of Public Health, Tehran University of Medical Sciences, Tehran, Iran; 4 Endocrine Research Center, Research Institute for Endocrine Sciences, Shahid Beheshti University of Medical Sciences, Tehran, Iran; Innsbruck Medical University, Austria

## Abstract

**Background:**

Data on incidence of coronary heart disease (CHD) is scarce in the Middle East and little is known about the contribution of known risk factors in this area.

**Methods:**

The incidence of CHD and the effect of modifiable risk factors were explored in 2889 men and 3803 women aged 30–74 years in the population based cohort of the Tehran Lipid and Glucose Study, during 1999–2010. Average population attributable fraction (aPAF) was calculated for any risk factor using direct method based on regression model.

**Results:**

The crude incidence rate in men was about twice that in women (11.9 vs. 6.5 per 1000 person-years). The aPAF of hypertension, diabetes, high total cholesterol and low-HDL cholesterol was 9.4%, 6.7%, 7.3% and 6.1% in men and 17%, 16.6%, 12% and 4.6% in women respectively. This index was 7.0% for smoking in men. High risk age contributed to 42% and 22% of risk in men and women respectively.

**Conclusions:**

The incidence in this population of Iran was comparable to those in the US in the seventies. Well known modifiable risk factors explained about 40% and 50% of CHD burden in men and women respectively. Aging, as a reflection of unmeasured or unknown risk factors, bears the most burden of CHD, especially in men; indicating more age-related health care is required.

## Introduction

Coronary heart disease (CHD) is one of the foremost causes of disease burden in developed and developing countries, however little data is available on sex-specific incidence of it in developing countries [1]. More than 40% of mortality in Tehran has been related to cardiovascular diseases (CVDs) [2]. Around 20% of adults aged 30 years and over in this capital city have symptoms or signs of CHD [3], and more than 70% of them had at least one CHD risk factor [4], a situation which needs to be explored further by determining the incidence of CHD and the importance of its risk factors. Population attributable fraction (PAF) is the most applicable index in public health which can be useful to assess the importance of risk factors. Previous studies have shown that common risk factors explain the majority of the burden of CHD [5], [6]; there remains substantial uncertainty about regional differences in PAF. There is lack of long term studies regarding incidence of CHD and PAF of traditional risk factors in Middle East population as to who will have the greatest increase in CHD risk factors [7]. The Tehran Lipid and Glucose study (TLGS) is a population based cohort from the Middle East which has previously been compared with the Framingham cohort regarding conventional cardiovascular risk factors [8]. This study with 10-years of follow-up allowed us to address the questions regarding the incidence of CHD and the PAF of its well-known modifiable risk factors reliably.

## Methods

### Study population

Tehran, the capital city of Iran is located 1500 Km north of the Persian Gulf. The TLGS is an ongoing population based cohort study initiated in 1999 to assess the associated risk factors of non-communicable diseases, including cardiovascular events, in an Iranian population [4]. The TLGS consists of 15005 individuals aged 3 years and over in district no.13 of Tehran, representative of Tehran population regarding age distribution; this district among other districts of Tehran has neither low nor high socioeconomic status. The sampling method has been described elsewhere [4], [9]. One part of the study population (n = 5630) has been involved in an educational program for life style modification [9].

**Figure 1:**
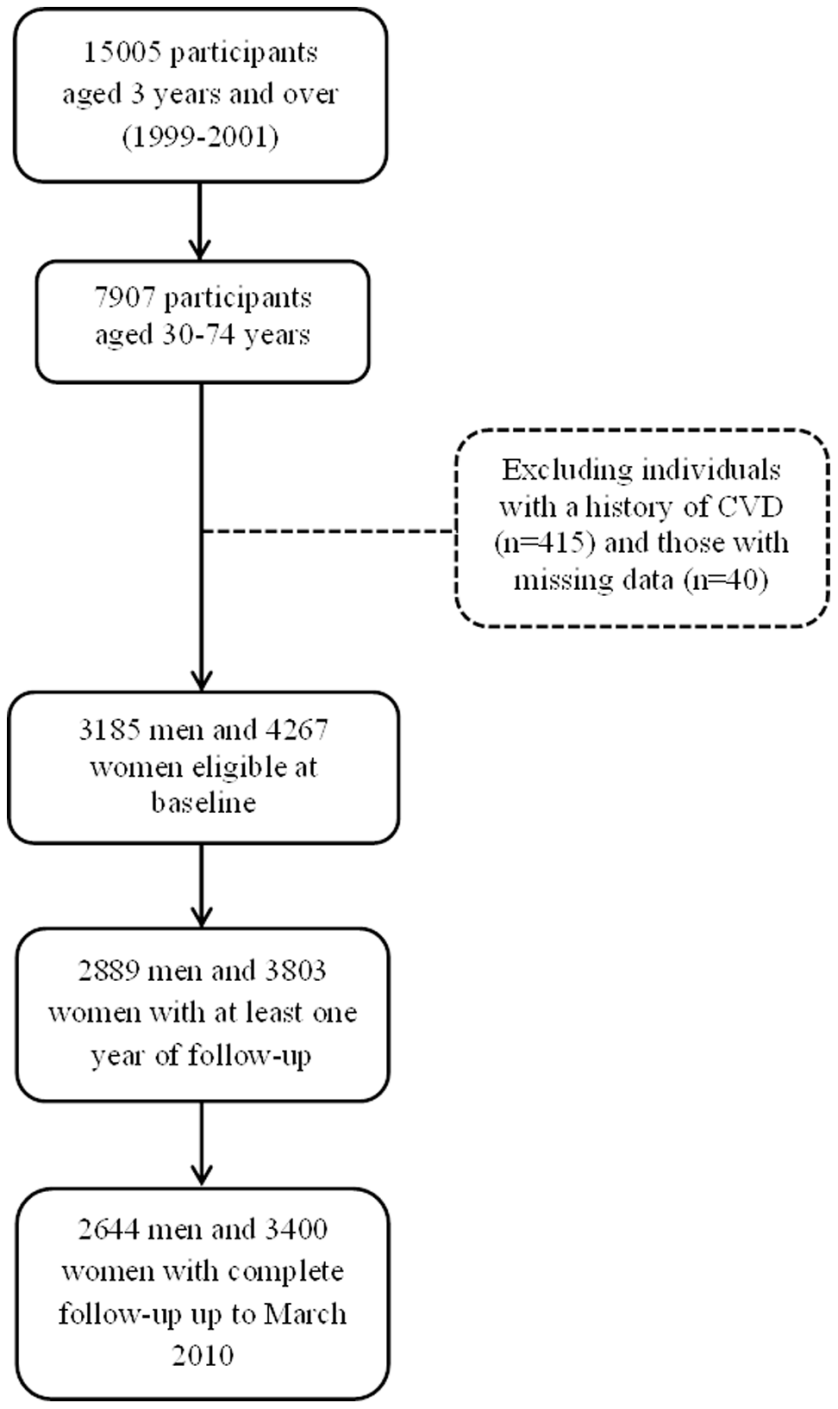
Flow chart of study population.

In the current study we considered all participants 30–74 years of age (n = 7907) and excluded individuals with a history of CVD (n = 415) and those with more than three missing variables (n = 40), leaving us with 3185 men and 4267 women. Participants were recruited during February 1999 to August 2001. Of them 2889 men and 3803 women had at least one year of follow-up with a median of 10.3 years and 2644 men and 3400 women had complete follow-up data up to 20 March 2010 (80% of eligibles) with a minimum of 8.6 and maximum of 11.1 years (Figure 1).

Informed written consent was obtained from all participants. The study was approved by the ethics committee of the Research Institute for Endocrine Sciences.

### Exposure variables

In the cross-sectional phase, from 1999 until 2001, data were gathered by means of interview, physical examination and laboratory measurements. The details of measurements have been described elsewhere [4]. In the current study we used Adult Treatment Panel III (ATPIII) recommendations [5] to define risk factors of CHD as follows: Hypertension, systolic blood pressure ≥140 mmHg or diastolic blood pressure ≥90 mmHg or taking hypertension medication; hypercholesterolemia, serum cholesterol ≥6.2 mmol/l (240 mg/dl) or taking cholesterol lowering medication; diabetes, fasting plasma glucose ≥7.0 mmol/l (126 mg/dl) or 2 hours post load glucose ≥11.1 mmol/l (200 mg/dl) or taking any medication for diabetes; low HDL cholesterol, HDL cholesterol <1.03 mmol/l (40 mg/dl); tobacco consumption, any tobacco product (cigarette, pipe, waterpipe) in the past or currently on a regular or occasional basis; family history of CVD, history of myocardial infarction or stroke or sudden cardiac death in a male first degree relative <55 years or female first degree relative <65 and the high risk age group, ages 45 and 55 years or higher in men and women respectively.

### Outcome variables

Outcome measurements have been described before [9], [10]. Briefly, the follow up survey was done annually by telephone calls, by which a trained nurse asked each participant about any medical event leading to hospitalization during the past year, and if positive, complementary data were collected by a trained physician using hospital records or if needed a home visit. In addition, in the case of mortality, data were collected from the death certificate, the forensic medicine report and if needed verbal autopsy. To confirm the diagnosis, an outcome committee reviewed all collected data. The committee consisted of an internist, a cardiologist, an endocrinologist, an epidemiologist, the physician who collected the data, and other experts invited as needed. In this study, CHD events as outcomes included cases of definite myocardial infarction (MI) diagnosed by electrocardiogram (ECG) and biomarkers, probable MI (positive ECG findings plus cardiac symptoms or signs but biomarkers showing negative or equivocal results), unstable angina pectoris (new cardiac symptoms or changing symptom patterns and positive ECG findings with normal biomarkers), angiography proven CHD and CHD death (any death due to CHD based on above criteria in hospital or sudden cardiac death from cardiac disease occurring less than or equal to 1 hr after onset of symptoms based on verbal autopsy documents outside of hospital). These are comparable with ICD10 rubric I20–I25.

### Statistical methods

Baseline characteristics regarding CHD risk factors are reported for men and women accompanied by a comparison using Chi-square or t-test. Incidence density of CHD was calculated per 1000 person-years of follow-up among participants with at least one year of follow-up and was standardized based on the age structure of Iranaian population in 2006 [11] and WHO standard population [12]. The crude incidence rate was done for high risk and low risk groups regarding each risk factor and compared between groups using Log Rank test for equality of event free survival. A Weibull accelerated failure time (AFT) model was conducted to estimate the effect of risk factors on the hazard of CHD and time to the event [13]. Weibull AFT model determines both effect measures of hazard ratio (HR) and time ratio (TR). When comparing one group to another, TR is a ratio of survival times corresponding to any fixed value of S(t). Time ratio <1.0 indicates acceleration of time to the cardiac event or shortening of event free survival time. We run Weibull model Because TR may disclose the effect of risk factors more tangibly, nevertheless, we run Cox regression analysis as well and showed that, given HR, the results were completely compatible. The appropriateness of the Weibull and AFT assumptions were evaluated using a plot of the log (−log) of Kaplan-Meier survival against the log of time [13].

The average PAF (aPAF) was calculated for any risk factors; aPAF is a multivariable adjusted attributable fraction which is directly calculated from individuals’ data using logistic regression and considers an average of all possible sequences for removal of risk factors in the community [14]. For this purpose, we used data of participants who had complete follow-up data and applied a STATA macro prepared for this purpose by Rückinger et al. [15]. A Hosmer-Lemeshow chi-square test was used to check the model fitness and chi-square <20 was considered suitable [16].

## Results

Table 1 illustrates the baseline characteristics and the prevalence of CHD risk factors at the beginning of study for men and women. For 2889 men and 3803 women at baseline, the mean (SD) ages were 47.5 (12.3) and 46.3 (11.4) respectively. Among modifiable risk factors, low HDL-C was the most prevalent risk factor in both genders, followed by smoking in men and hypercholesterolemia in women. The frequencies of all risk factors differed significantly between men and women (all p<0.05), although means of systolic blood pressure and fasting plasma glucose did not. Comparing individuals who were followed up to the end of the study with others revealed that they were less smoker (44.4% vs. 52.2% in men and 6.4% vs. 9.4% in women, p<0.05 in both gender) and younger just in women (46.1 vs. 47.8 years, p = 0.01), but there were no other significant differences in their baseline characteristics.

**Table 1:** Baseline characteristics of the study population regarding coronary heart disease risk factors.

	Men (n = 2889)	Women (n = 3803)	P-value^a^
**Variables**			
** Age (years)**	47.5 (12.3)	46.3 (11.4)	<0.001
** Systolic blood pressure, mmHg**	121.6 (19.1)	121.9 (20.4)	0.568
** Diastolic blood pressure, mmHg**	78.9 (11.2)	79.5 (10.7)	0.030
** Fasting plasma glucose, mmol/l**	5.57 (1.82)	5.63 (2.14)	0.207
** 2 hours post load glucose mmol/l** b	5.82 (1.76)	6.29 (1.57)	<0.001
** Total cholesterol, mmol/l**	5.44 (1.11)	5.74 (1.22)	<0.001
** HDL cholesterol, mmol/l**	0.99 (0.24)	1.16 (0.29)	<0.001
** Hypertension medication, n**	128 (4.4%)	421 (11.1%)	<0.001
** Diabetes medication, n**	97 (3.4%)	210 (5.5%)	<0.001
** Anti-lipid medication, n**	63 (2.2%)	189 (5.0%)	<0.001
** Defined risk factors** c			
** High risk age**	1510 (52.3%)	980 (25.8%)	<0.001
** Family history of cardiovascular diseases**	405 (14.0%)	698 (18.4%)	<0.001
** Smoking**	1303 (45.1%)	255 (6.7%)	<0.001
** Hypertension**	661 (22.9%)	1049 (27.6%)	<0.001
** Diabetes Mellitus**	345 (11.9%)	534 (14.0%)	0.012
** High total cholesterol**	657 (22.7%)	1267 (33.3%)	<0.001
** Low HDL cholesterol**	1915 (66.3%)	1423 (37.4%)	<0.001

Data are shown as mean (SD) or frequency (%).

a For difference between men and women based on t-test for continuous and chi-square test for binary variables.

b In participants without any diagnosed diabetes.

c Definition of risk factors is according to the Adult Treatment Panel III. All definitions are similar in men and women except high risk age; it is ages 45 or higher in men and ages 55 or higher in women.

After 26942 and 36533 person-years of follow-up, 320 and 236 first CHD events including 42 and 24 fatal CHD, 64 and 27 nonfatal myocardial infarction, 142 and 107 angiography proven CHD and 72 and 78 unstable angina occurred in men and women respectively. The crude incidence rate of CHD was calculated 11.9 (95% CI, 10.6–13.2) per 1000 person-years in men and 6.5 (5.7–7.3) per 1000 person-years in women. The age-standardized incidence rate based on Iranian population was 10.5 (9.3–11.6) and 6.1 (5.3–6.9) per 1000 person-years in men and women respectively; these rates based on WHO standard population were 12.2 (10.8–13.5) and 7.4 (6.4–8.3). The crude incidence rate of CHD in high and low risk individuals, regarding different risk factors, is shown in Table 2. Except low HDL cholesterol in both genders, family history of CVD in men and smoking in women, other defined risk factors had a significant effect on the incidence of CHD in univariate analysis (Table 2).

**Table 2:** Incidence density (95% CI)^a^ for coronary heart disease regarding risk factors at baseline.

Risk factor^b^	Risk factor –	Risk factor +	P-Value^c^
**Men**			
** High risk age**	4.2 (3.2–5.4)	19.5 (17.3–22.0)	<0.001
** Family history of cardiovascular diseases**	11.4 (10.2–12.9)	14.5 (11.1–18.9)	0.107
** Smoking**	10.4 (8.9–12.2)	13.7 (11.7–15.9)	0.012
** Hypertension**	8.8 (7.6–10.1)	23.2 (19.6–27.5)	<0.001
** Diabetes Mellitus**	9.6 (8.4–10.9)	31.4 (25.5–38.7)	<0.001
** High total cholesterol**	9.7 (8.5–11.1)	19.5 (16.2–23.4)	<0.001
** Low HDL cholesterol**	10.9 (9.0–13.3)	12.4 (10.8–14.1)	0.283
**Women**			
** High risk age**	3.3 (2.7–4.0)	16.4 (13.9–19.3)	<0.001
** Family history of cardiovascular diseases**	5.6 (4.9–6.6)	10.2 (8.0–12.9)	<0.001
** Smoking**	6.3 (5.5–7.2)	8.5 (5.5–13.2)	0.196
** Hypertension**	3.4 (2.8–4.2)	14.9 (12.6–17.5)	<0.001
** Diabetes Mellitus**	4.0 (3.4–4.8)	22.9 (18.9–27.6)	<0.001
** High total cholesterol**	3.8 (3.1–4.7)	11.9 (10.1–14.0)	<0.001
** Low HDL cholesterol**	6.2 (5.2–7.3)	6.9 (5.7–8.5)	0.367

a Incidence density per 1000 person-years among participants with at least one year of follow-up (2889 men and 3803 women).

b Definition of risk factors is according to the Adult Treatment Panel III.

c Based on Log Rank test for equality of event free survival between groups.

**Figure 2:**
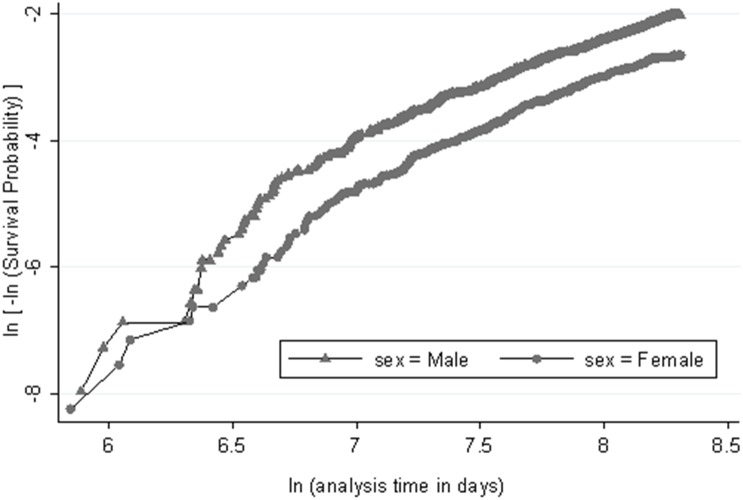
Log negative log Kaplan–Meier survival against the log of time (in days). Straight lines suggest a reasonable assumption for Weibull model in both genders and parallel lines for men and women indicates reasonable proportional hazard assumption.

Straight line in the log (–log) plot of Kaplan-Meier survival against the log of time indicated that the Weibull model assumption is reasonable in both genders (Figure 2). Repeating this plot for any risk factors showed reasonable parallel lines for individuals with and without risk factors, indicating reasonable AFT and proportional hazard assumption (data not shown). Multivariable analysis based on the Weibull model showed a shape parameter (*p*) of 1.7 (95% CI, 1.5–1.8) in men and 1.7 (1.5–2.0) in women indicating that the hazard increases over the time; it also indicated a borderline significant effect of low HDL cholesterol on CHD outcome and significant effects for other risk factors, except smoking in women (Table 3). High risk age revealed a HR of 3.9 (2.9–5.3) in men and 2.7 (2.0–3.6) in women. Among modifiable risk factors, diabetes had the greatest effect on CHD incidence with a reduction of 35% and 49% in the CHD free survival time in men and women respectively (TR 0.65, 95% CI 0.55–0.76, in men and 0.51, 0.43–0.61, in women). Low HDL cholesterol had the least effect on time to CHD event; it decreased CHD free survival time by 10% in men and 12% in women (TR 0.90, 0.78–1.04, in men and 0.88, 0.75–1.02 in women). Given HR, the results of Weibull model were completely compatible with results from Cox proportional hazard model (Table S1).

**Table 3:** Hazard ratio, survival time ratio, odds ratio and population attributable fraction (PAF) for coronary heart disease risk factors.

Risk factors	Hazard Ratio(95% CI)^a^	Time Ratio (95%CI)^a^ ^b^	Odds Ratio(95% CI)^c^	Average PAF(%)^c^ ^d^
**Men**				
** High risk age**	3.9 (2.9–5.3)	0.44 (0.36–0.54)	4.0 (3.0–5.6)	42.0
** Family history of** **cardiovascular diseases**	1.4 (1.1–1.9)	0.81 (0.68–0.97)	1.4 (1.0–2.0)	2.5
** Current smoking**	1.6 (1.2–2.0)	0.76 (0.66–0.88)	1.7 (1.3–2.2)	7.0
** Hypertension**	1.8 (1.4–2.2)	0.71 (0.61–0.82)	1.9 (1.4–2.4)	9.4
** Diabetes mellitus**	2.0 (1.6–2.6)	0.65 (0.55–0.76)	2.1 (1.6–2.9)	6.7
** High total cholesterol**	1.6 (1.3–2.1)	0.74 (0.65–0.86)	1.7 (1.3–2.3)	7.3
** Low HDL cholesterol**	1.2 (0.9–1.5)	0.90 (0.78–1.04)	1.2 (0.9–1.6)	6.1
**Women**				
** High risk age**	2.7 (2.0–3.6)	0.56 (0.47–0.67)	3.0 (2.2–4.1)	22.0
** Family history of** **cardiovascular diseases**	1.7 (1.3–2.3)	0.73 (0.62–0.86)	1.9 (1.4–2.6)	6.8
** Current smoking**	1.2 (0.6–2.4)	0.89 (0.60–1.30)	1.3 (0.6–2.6)	
** Hypertension**	2.1 (1.6–2.8)	0.65 (0.54–0.77)	2.3 (1.7–3.1)	17.0
** Diabetes mellitus**	3.2 (2.4–4.2)	0.51 (0.43–0.61)	3.5 (2.6–4.8)	16.6
** High total cholesterol**	1.6 (1.2–2.2)	0.75 (0.64–0.89)	1.7 (1.3–2.3)	12.0
** Low HDL cholesterol**	1.3 (1.0–1.6)	0.88 (0.75–1.02)	1.3 (1.0–1.7)	4.6

a For study population of 2889 men and 3803 women with at least one year of follow-up.

b Time ratio <1.0 indicates acceleration of time to the cardiac event or shortening of event free survival time.

c For study population of 2645 men and 3400 women with a complete follow-up. Hosmer-Lemeshow chi-square of 11.0 in men and 6.7 in women indicated appropriate logistic models.

d A multivariable adjusted attributable fraction which is directly calculated from individuals’ data using logistic regression and considers an average of all possible sequences for removal of risk factors in the community.

Few individuals had not a complete follow-up (245 men and 403 women) and considering those with complete follow-up, no sensible changes in the prevalence of risk factors at the baseline was observed. Odds ratios calculated by logistic regression were compatible with hazard ratios accrued by Weibull model (Table 3). Hosmer-Lemeshow goodness of fit test showed chi-square = 11.0 in men and chi-square = 6.7 in women indicating appropriate logistic models. Table 3 also shows aPAF based on logistic regression in subjects with complete follow-up. The highest aPAF was due to high risk age; however the rank of aPAFs among modifiable risk factors revealed that hypertension in men and hypertension and diabetes in women had the highest aPAF. The sum of calculated aPAFs was 81% (including 36.5% for modifiable risk factors) in men and 79% (including 50.2% for modifiable risk factors) in women.

## Discussion

This study determined the 10-year incidence of coronary heart disease as well as a quantitative relationship between CHD and conventional risk factors in a population based study in Iran as a middle income country in the Middle East. Computing aPAF for defined risk factors enabled us to determine the burden of these factors for CHD in our population. We determined how much of this burden belongs to the modifiable risk factors.

The incidence rate in men was almost twice that of women (WHO age standardized of 12.2 vs. 7.4 per 1000 person-years). As a comparison, this incidence of CHD is approximately equal to that of the US during 1971–1989 (11 and 6.4 per 1000 person-years for men and women respectively) [17], however seems lower than that in Northern Europe and higher than Southern Europe [18]. The incidence of CHD in the East Asia during the last decade was much lower than that observed in our population. A study from Beijing, China showed an age-standardized incidence of 2.2 in men and 1.2 in women per 1000 persons [19]. Another study reported this finding for Japanese aged 40–69 years, equal to 1.0 and 1.8 per 1000 persons for men and women respectively [20]. The total crude incidence rate of CHD in Turkey, our neighbor country in Middle East region, among 30–74 years old individuals, has been reported to be 17.2 per 1000 persons/year [21]. The adjusted incidence rate of CHD in other part of Iran, three districts in the center of Iran including Isfahan, Najafabad, and Arak, is 11.7 and 8.9 per 1000 person years in men and women respectively [22], a little higher than adjusted incidence rate in Tehran (10.5 and 6.1 respectively) bearing in mind, that incidence is due to both urban and rural areas and our incidence is due to just urban area.

Our study showed that conventional risk factors explain about 80% of CHD events in our population with some differences in men and women. CHD incidence rates in both genders figures out the several main points which need to be considered: First of all, as high risk age was the most important unmodifiable risk factor and since demographic transition of aging has begun in Iran [23], aging could have a major effect on the occurrence of CHD at this time. Several studies support this finding [24], [25], [26]. Odden et al., pointed out that with no extensive changes in risk factors or clinical care, the aging of the US population will result in a considerable increase in CHD incidence, prevalence and mortality [27]. The risk of aging for CHD was higher in men than women. Aging might be a surrogate of previous hazardous exposures throughout life; men are more exposed to these exposures due to their occupation and lifestyle status. These exposures (like air pollution, stress, etc.) are not usually measured or known. On the other hand increasing age in women is along with increasing the risk of menopause. To be more comparative, we calculated HR for 1-SD change of age and found nearly the same HR in both gender (1.9, 95% CI: 1.7–2.1 in men and 2.1, 95% CI: 1.7–2.4 in women, adjusted for other risk factors).The aPAF of high risk age in men was about two folds that of women which is partly due to the threshold considered for definition of high risk age (45 years for men and 55 for women) [5], resulted in a higher prevalence of this risk factor in men than in women.

Secondly, our finding established the effect of modifiable cardiovascular risk factors which first came to light from the Framingham heart study. We compared the relative risk of different risk factors between Framingham and TLGS cohort in detail before [8]. Through the decades, the normal value for standard risk factors such as high blood pressure and total cholesterol had changed, and new risk factors were added to the primary list; however the impacts of classic risk factors are as important as ever and much of the mortality and morbidity as a consequence of atherosclerosis are linked to these factors [25]. In our study, the most prevalent modifiable risk factors were low HDL cholesterol and smoking in men and low HDL cholesterol followed by high total cholesterol in women; however the most influential risk factors for CHD were diabetes and hypertension in both genders. The PAF, as a reasonable way to surrogate the prevalence and relative risk of a risk factor showing its burden, indicated that controlling hypertension in men and, hypertension and diabetes in women would be the priorities for CHD prevention strategy. Nevertheless, only lower than 50% of CHD burden in men was modifiable and the most was due to aging. Sniderman and Furberg [28] interpreted age as some time-related effects of disintegration which affect all of us versus time-related effects of modifiable risk factors that affect some of us more than others and the methods used to predict risk cannot take into account this distinction. Additionally, we believe that age consists of residual risks due to modifiable risk factors in their value below the threshold of high risk definition.

Smoking was a significant risk factor in men causing an aPAF of 7%, but being an uncommon practice in women and was not considered a major risk for them, the point which had previously been shown among Turks as well [29]. Diabetes had an aPAF of 6.7% in men and 16.6% in women; since, an almost two fold prevalence is predicted in 2030 [30], needs to act accordingly promptly. The higher risk for diabetes among women was compatible with our previous reports and those of other large population studies [31], [32], [33].

In a national survey among Iranian adult population, it was shown that about 25% of Iranians aged 25–64 years had hypertension; among hypertensive patients, only 34% were aware of their hypertension and 25% were taking medications; and of these treated subjects, only 24% had a controlled blood pressure [34]. Furthermore, in the current study, in both genders and especially in women, we highlighted hypertension as a most important modifiable risk factor for incident CHD. These findings showed the emergent need to develop national policies to improve prevention, detection, and treatment of hypertension in Iran.

The borderline significance of risk of low HDL-C among our population might be related to the inappropriate cut off point or lack of variation of this risk factor among our population [10]. Considering HDL-C in tertiles, showed an increasing protective effect of HDL-C on CHD (p = 0.01 and p = 0.09 for trend in men and women respectively, data not shown), however treating HDL-C as a binary variable, to calculate aPAF, revealed a minor effect (Table 3); nevertheless it made a reasonable aPAF because of high prevalence of low HDL-C especially in men.

The PAF could differ from one setting to another, due to various methods and the different prevalent risk factors in each geographical area. A comparative study showed that CHD event attributable to major risk factors was almost similar between Argentina and the US, except for high SBP in men (PAF 28.8% in Argentina vs 21.2% in US) [35]. Using the aPAF method Rückinger et al. showed that 90% of CVD incidence can be attributed to high age, male sex, hypertension, high total cholesterol, low HDL, smoking and diabetes [15].

As strengths, this study benefitted from a prospective cohort design with small number of lost to follow up, a good fitness of the statistical model and a sensitive method for calculating PAF. To be consistent with other studies, we only used definite risk factors of CHD based on ATPIII definitions [5]; however someone may criticize the limited number of risk factors analyzed in predicting the outcome, specifically, the absence of an adiposity measure is conspicuous and relevant which may have an independent effect on CHD beyond the effect of mediators i.e. hypertension, diabetes and hyperlipidemia [36]. Further analysis showed that general obesity (BMI≥30), adjusted for other risk factors, did not have any significant independent effect on CHD, however central obesity (waist ≥95 cm based on national definition [37]) had a significant HR of 1.3 (95% CI: 1.1–1.7) in men and a borderline significant HR of 1.3 (1.0–1.7) in women. Adding central obesity to the direct estimation of PAF, showed an average PAF of 6.2% and 5.5% in men and women respectively but did not increase the sum of PAFs in men and improved the sum of PAFs just for 2 percent in women; on the other word, adding central obesity to the direct estimation of PAF decreased the PAF of others. Triglyceride as another lipid measure did not have an independent effect on CHD and there was no data regarding inflammation-related markers such as CRP and fibrinogen.

This study might fail to include all CHD events in the target population which could be described by different types of private and public healthcare systems in Iran. Also like other cohort studies, our study may be affected by regression dilution bias. Tehran is a large urban area and extending the results to other urban and rural area is limited.

To conclude, Tehran has a modest incidence of CHD among other regions in the world and aging bears the most burden of CHD especially in men. Regarding demographic transition in the middle income countries, devotion to age-related health care is required like high income ones. Well-known modifiable risk factors as the necessary preventable components of causal diagram for CHD, contribute to about 40% and 50% of the CHD risk in men and women respectively; hypertension for both genders and diabetes in women, are the most sensible priorities for preventive strategies in our population.

## Supporting Information

Table S1 **Hazard ratio for coronary heart disease risk factors based on Cox proportional hazard model.**
(DOCX)
