# Supplementary material for: The Incidence of Coronary Heart Disease and the Population Attributable Fraction of Its Risk Factors in Tehran: A 10-Year Population-Based Cohort Study
Source: PLoS One. 2014 Aug 27;9(8):e105804. doi: 10.1371/journal.pone.0105804 (PMC4146560; doi:10.1371/journal.pone.0105804)
Supplement: Table S1 — Hazard ratio for coronary heart disease risk factors based on Cox proportional hazard model. (DOCX) [file pone.0105804.s001.docx]

**Table S1: Hazard ratio for coronary heart disease risk factors based on Cox proportional hazard model**

| **Risk factors** | | **Hazard Ratio**  **(95% CI) ^a^** |  |  |  |
| --- | --- | --- | --- | --- | --- |
| **Men** | | | | | |
|  | **High risk age** | 3.9 (2.9-5.2) |  |  |  |
|  | **Family history of cardiovascular diseases** | 1.4 (1.1-1.9) |  |  |  |
|  | **Current smoking** | 1.6 (1.2-2.0) |  |  |  |
|  | **Hypertension** | 1.8 (1.4-2.2) |  |  |  |
|  | **Diabetes mellitus** | 2.0 (1.6-2.6) |  |  |  |
|  | **High total cholesterol** | 1.6 (1.3-2.1) |  |  |  |
|  | **Low HDL cholesterol** | 1.2 (0.9-1.5) |  |  |  |
| **Women** | | | | | |
|  | **High risk age** | 2.7 (2.0-3.6) |  |  |  |
|  | **Family history of cardiovascular diseases** | 1.7 (1.3-2.3) |  |  |  |
|  | **Current smoking** | 1.2 (0.6-2.4) |  |  |  |
|  | **Hypertension** | 2.1 (1.6-2.8) |  |  |  |
|  | **Diabetes mellitus** | 3.2 (2.4-4.1) |  |  |  |
|  | **High total cholesterol** | 1.6 (1.2-2.1) |  |  |  |
|  | **Low HDL cholesterol** | 1.3 (1.0-1.6) |  |  |  |

**^a^** For study population of 2889 men and 3803 women with at least one year of follow-up.
